# Surgical site infection surveillance in Ireland: a snapshot of current practice

**DOI:** 10.1016/j.infpip.2026.100567

**Published:** 2026-07-11

**Authors:** Emily Glynn, Sinead Hurley, Lauren Webster, Clare Rock, Karina O'Connell, Sinead O'Donnell, Eimear Brannigan

**Affiliations:** a Department of Clinical Microbiology, Beaumont Hospital, Dublin, Ireland; b Department of Clinical Microbiology, The Royal College of Surgeons in Ireland (RCSI), Dublin, Ireland; c HSE Antimicrobial Resistance and Infection Control (AMRIC) Team, Health Service Executive, Dublin, Ireland; d Department of Clinical Microbiology, St Vincent's University Hospital, Dublin, Ireland

## Abstract

**Introduction:**

Surgical site infection (SSI) surveillance is an essential element of an effective infection prevention and control programme. Ireland’s national SSI surveillance programme is in its infancy, commencing in 2023 with surveillance data collected post hip fracture surgery. However, many hospitals have already implemented local SSI surveillance activities. The aim of this survey was to establish the extent and structure of these programmes.

**Methods:**

A cross-sectional online survey was distributed to consultant clinical microbiologists via the Irish Society of Clinical Microbiology mailing list (January–March 2025), including quantitative and optional qualitative components.

**Results:**

Responses were received from 37 hospitals, including both adult and paediatric centres and public (*N* = 32) and private institutions (*N* = 5). Public hospitals were well represented (32/47; 68% response rate). Twenty-one hospitals reported established SSI surveillance programmes. Commonly monitored procedures included elective hip and knee replacement surgery (*N* = 10), cholecystectomy (*N* = 8), cardiac surgery (*N* = 7), spinal surgery (*N* = 6), and hip fracture surgery (*N* = 6). Twelve sites employed dedicated SSI clinical nurse specialists. Where programmes existed, 48% had an SSI committee. The most common approach was prospective case surveillance (*N* = 14). Ten sites performed active postdischarge surveillance, using a combination of phone calls (*N* = 9), postdischarge patient questionnaires (*N* = 3), and text messages (*N* = 2). Challenges reported include provision of dedicated staff, Information Technology systems, governance structures, and postdischarge surveillance.

**Conclusion:**

SSI surveillance in Ireland is variable and resource-intensive, reflecting local adaptation without national standardisation. These findings highlight the need for co-ordinated national frameworks, investment in staffing and digital infrastructure, and integration of existing systems to support sustainable expansion of Ireland’s national programme.

## Introduction

Surgical site infection (SSI) is one of the most common healthcare-associated infections and remains a significant cause of postoperative morbidity, prolonged length of hospital stays, and increased healthcare costs. Surveillance is a key component of effective infection prevention and control (IPC) programmes, enabling measurement of incidence, identification of trends, and evaluation of quality improvement initiatives [1]. However, accurately estimating the true burden of SSI remains challenging due to diagnostic uncertainty and heterogeneity in surveillance methodologies.

This survey aimed to describe current SSI surveillance practices across Irish hospitals and identify variation to inform the development of a co-ordinated national programme.

SSI is defined as an infection occurring within 30 days postoperatively or within 90 days if a prosthesis is inserted and may be classified as superficial incisional, deep incisional, or organ/space infection [2]. Surveillance methods vary. Active prospective surveillance, enabling real-time case identification, is considered the optimal approach but may not be feasible where local resources are limited [3,4].

While other countries such as the UK have well-established national SSI surveillance programmes, Ireland’s programme is still in development. The Republic of Ireland operates a mixed public–private healthcare system, with public care delivered through the Health Service Executive (HSE). Establishing a national SSI surveillance system is a key objective of the HSE Antimicrobial Resistance and Infection Prevention Control (AMRIC) Action Plan [5]. In 2023, national SSI surveillance following hip fracture surgery commenced in sixteen public hospitals in collaboration with the National Office of Clinical Audit (NOCA) and the Irish Hip Fracture Database (IHFD).

Further expansion of the Irish national SSI surveillance programme is planned. However, many hospitals have independently established local SSI surveillance systems. These often operate in isolation, without standardised approaches to data collection, benchmarking, or reporting, limiting opportunities for system-wide learning and improvement. Participation in benchmarking networks has been associated with reductions in SSI rates [6], underscoring the value of co-ordinated national efforts.

## Methods

A cross-sectional online survey was conducted to explore local SSI surveillance practices in Irish hospitals. The survey was distributed to consultant clinical microbiologists through the Irish Society of Clinical Microbiology (ISCM) consultant mailing list. All clinical microbiology consultants working in public or private healthcare institutions in Ireland are eligible for ISCM membership and therefore represented the target population. A total of 47 public and 17 private hospitals were eligible for inclusion. One response per hospital site was requested to avoid duplication.

The questionnaire was developed by the AMRIC team based on key components of SSI surveillance identified in the literature and national programme priorities and was piloted internally to ensure clarity and relevance prior to dissemination. The survey was hosted on a secure online platform (SmartSurvey) and responses were accepted from January to March 2025. The survey invitation included an introductory statement describing the purpose, voluntary participation, and data confidentiality. Completion and submission of the survey were considered as implied consent.

The questionnaire included both closed-ended questions and optional open-ended questions to capture both quantitative data and contextual detail. No identifiable personal data were collected, and hospital sites were anonymised. Data were stored on password-protected systems and analysed in aggregate. Quantitative data were summarised descriptively using counts and proportions. Associations between programme status and categorical variables (dedicated SSI personnel and electronic patient record availability) were examined using Fisher’s exact test, with a *P* value <0.05 considered statistically significant. Free-text responses were analysed thematically by the AMRIC team through an iterative review to identify key recurring themes.

## Results

### Participation and coverage

Responses were received from 37 hospitals, including both adult (*N* = 35) and paediatric centres (*N* = 2) and public (*N* = 32) and private institutions (*N* = 5). These represented 37 of 64 eligible hospitals (58%). Overall, 21 of 37 (57%) hospitals reported having established SSI surveillance programmes including all private hospitals. Hospital characteristics and SSI programme structures are summarised in Table I, with detailed surveillance methodologies presented in Table II.

**Table I:** Hospital characteristics and SSI programme structure

Hospital ID^a^	Hospital type	Patient population	Procedures under SSI surveillance	SSI CNS	SSI committee	EPR availability
H1	Public	Adult	5	Yes	Yes	No
H2	Public	Adult	3	Yes	Yes	No
H3	Public	Adult	1	No	No	No
H4	Public	Paediatric	1	No	Yes	No
H5	Public	Adult	2	No	No	No
H6	Public	Adult	3	Yes	No	No
H7	Public	Adult	1	No	No	Yes
H8	Public	Adult	1	No	No	Yes
H9	Public	Paediatric	2	Yes	No	No
H10	Public	Adult	1	Yes	Yes	No
H11	Public	Adult	3	Yes	Yes	No
H12	Public	Adult	5	Yes	Yes	No
H13	Public	Adult	11	No	Yes	No
H14	Public	Adult	1	Yes	No	Yes
H15	Public	Adult	2	No	No	Yes
H16	Public	Adult	1	Yes	No	No
H17	Private	Adult	3	No	No	No
H18	Private	Adult	7	Yes	Yes	No
H19	Private	Adult	7	Yes	Yes	No
H20	Private	Adult	6	Yes	Yes	No
H21	Private	Adult	4	No	No	Yes

This table presents characteristics of hospitals with SSI surveillance programmes (*N* = 21).

SSI, surgical site infection; CNS, clinical nurse specialist; EPR, electronic patient record.

a H1–H21: Individual hospitals anonymised for confidentiality.

**Table II:** SSI surveillance characteristics

Hospital ID^a^	Case definition	Surveillance approach	Follow-up duration	Post-discharge surveillance	Method
H1	CDC	Prospective	30/90 days	Passive	Readmission
H2	CDC	Prospective	30/90 days	Active	Telephone
H3	ECDC	Prospective	30 days	Active	Telephone, text, questionnaire
H4	Combined	Prospective	30 days/>1 year	Active	Telephone, text
H5	CDC	Prospective	30 days/1 year	Active	Telephone, outpatient review
H6	CDC	Prospective	30/90 days	Active	Telephone
H7	ECDC	Retrospective	30 days	Passive	Readmission
H8	ECDC	Prospective	30/90 days	Passive	Readmission
H9	CDC	Prospective	30/90 days	Active	Physician contact
H10	ECDC	Retrospective	Inpatient	No	
H11	ECDC	Prospective	30/90 days	Active	Questionnaire, telephone
H12	CDC	Prospective	30/90 days	Active	Questionnaire, telephone, physician contact
H13	Combined	Combination	30/90 days	Passive	Readmission
H14	ECDC	Combination	30 days	Active	Telephone
H15	ECDC	Combination	30 days	Active	Telephone
H16	ECDC	Retrospective	30 days	No	
H17	Combined	Prospective	30/90 days	Passive	Readmission
H18	CDC	Prospective	30/90 days	Passive	Readmission
H19	CDC	Prospective	30/90 days	Passive	Readmission
H20	CDC	Prospective	30/90 days	Passive	Readmission
H21	ECDC	Combination	30/90 days	Passive	Readmission

This table presents surveillance methodologies across hospitals with SSI programmes (*N* = 21).

SSI, surgical site infection; CDC, Centers for Disease Control; ECDC, European Centre for Disease Prevention and Control.

a H1–H21: Individual hospitals anonymised for confidentiality.

### Surveillance characteristics

**Figure 1:**
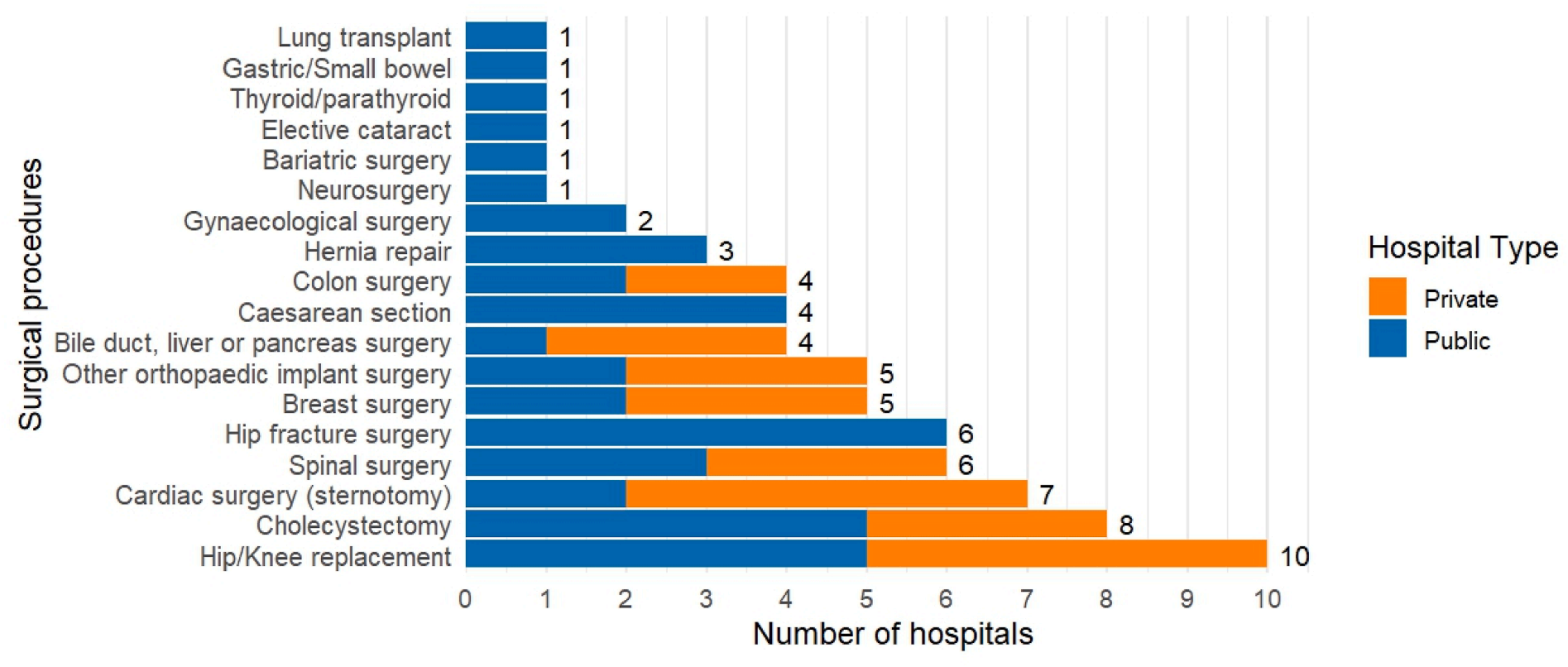
Surgical procedures monitored for SSI development. SSI, surgical site infection.

Procedures most frequently monitored included elective hip and knee replacement surgery (*N* = 10; 48%), cholecystectomy (*N* = 8; 38%), cardiac surgery (*N* = 7; 33%), spinal surgery (*N* = 6; 29%), and hip fracture surgery (*N* = 6; 29%) (Figure 1). SSI diagnosis was multi-disciplinary in 19 of 21 sites (90%). Case definitions varied; 9 of 21 (43%) applied the Centers for Disease Control (CDC) definition [4], 9 of 21 (43%) used the European Centre for Disease Prevention and Control (ECDC) [2], and 3 of 21 (14%) used combined approaches.

### Surveillance methods

Prospective surveillance was most common (*N* = 14; 67%), with retrospective (*N* = 3; 14%) and combined approaches (*N* = 4; 19%) also reported. Most sites (19/21; 91%) conducted postdischarge surveillance to 30 or 90 days. Postdischarge monitoring was passive in 9 of 21 sites (43%) and active in 10 of 21 sites (48%). Active methods were most commonly telephone follow-up (*N* = 9; 90%), followed by questionnaires (*N* = 3; 30%), general practitioner/surgeon contact (*N* = 2; 20%), text messaging (*N* = 2; 20%), and outpatient review (*N* = 1; 10%).

### Governance and resources

A dedicated SSI clinical nurse specialist (CNS) was used in 12 of 21 sites (57%). Additional surveillance support was provided by IPC nurses (2/21; 10%), surveillance scientists (2/21; 10%), NOCA audit co-ordinators (2/21; 10%), and neurosurgical CNS (1/21; 5%). Surveillance was performed without formal staff allocation in 4 of 21 sites (19%), while data collection was multi-disciplinary in 8 of 21 sites (38%). Only 10 of 21 (48%) sites had an SSI committee, and 5 of 21 (24%) reported electronic patient records supporting surveillance.

### Challenges and Future priorities

Common challenges reported included inadequate staffing, Information Technology (IT) infrastructure, engagement from surgical colleagues, governance structures, and postdischarge surveillance. All respondents (37/37) expressed a desire to expand surveillance, with priority areas including caesarean section and elective orthopaedic and colorectal surgery.

## Discussion

This national survey provides the first comprehensive overview of local SSI surveillance activities across Ireland, representing an important step as the country advances towards a national programme.

Over half of hospitals (57%, 21/37), reported local SSI surveillance, including 50% (16/32) of public and all private hospital respondents. Public hospitals were well represented (32/47; 68% response rate), while the response rate from private hospitals was lower – 5 of 17 (29%) – providing a meaningful national snapshot. These findings should be interpreted cautiously, as reported variation may reflect response bias and differences in surveillance definitions and reporting practices, rather than true differences in implementation. As the HSE has governance over public hospitals, these will form the foundation of a national SSI surveillance system.

Marked variability in surveillance methodology was evident. Hospitals used differing case definitions, staffing structures, data collection methods, and follow-up periods, reflecting local adaptation to resources and strategic priorities. This heterogeneity introduces diagnostic uncertainty and limits comparability of SSI rates across institutions. Such variation is consistent with international experience [7] and highlights the need for standardised national definitions and processes to enable meaningful benchmarking.

**Figure 2:**
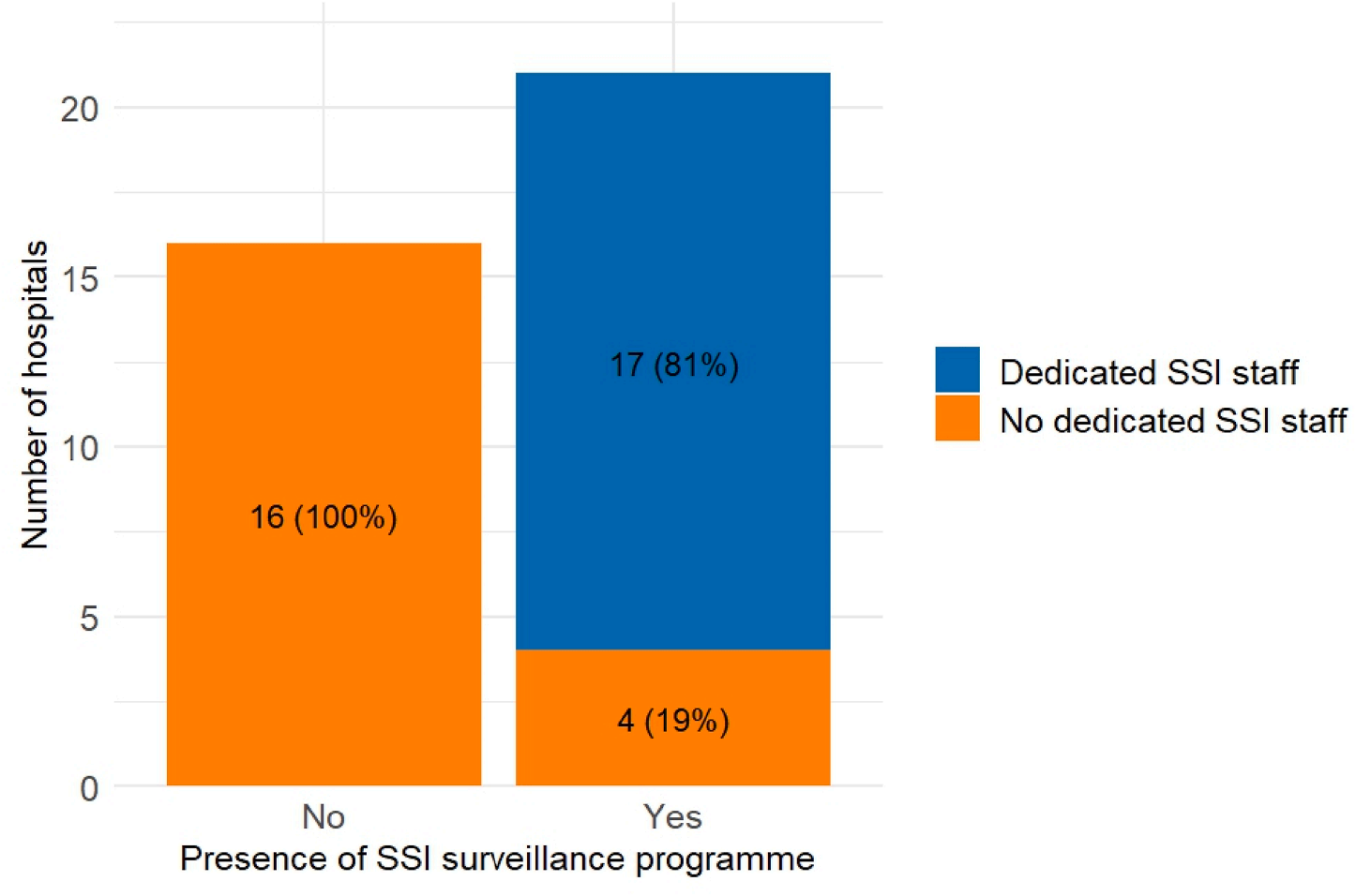
Personnel support by SSI surveillance programme status. SSI, surgical site infection.

**Figure 3:**
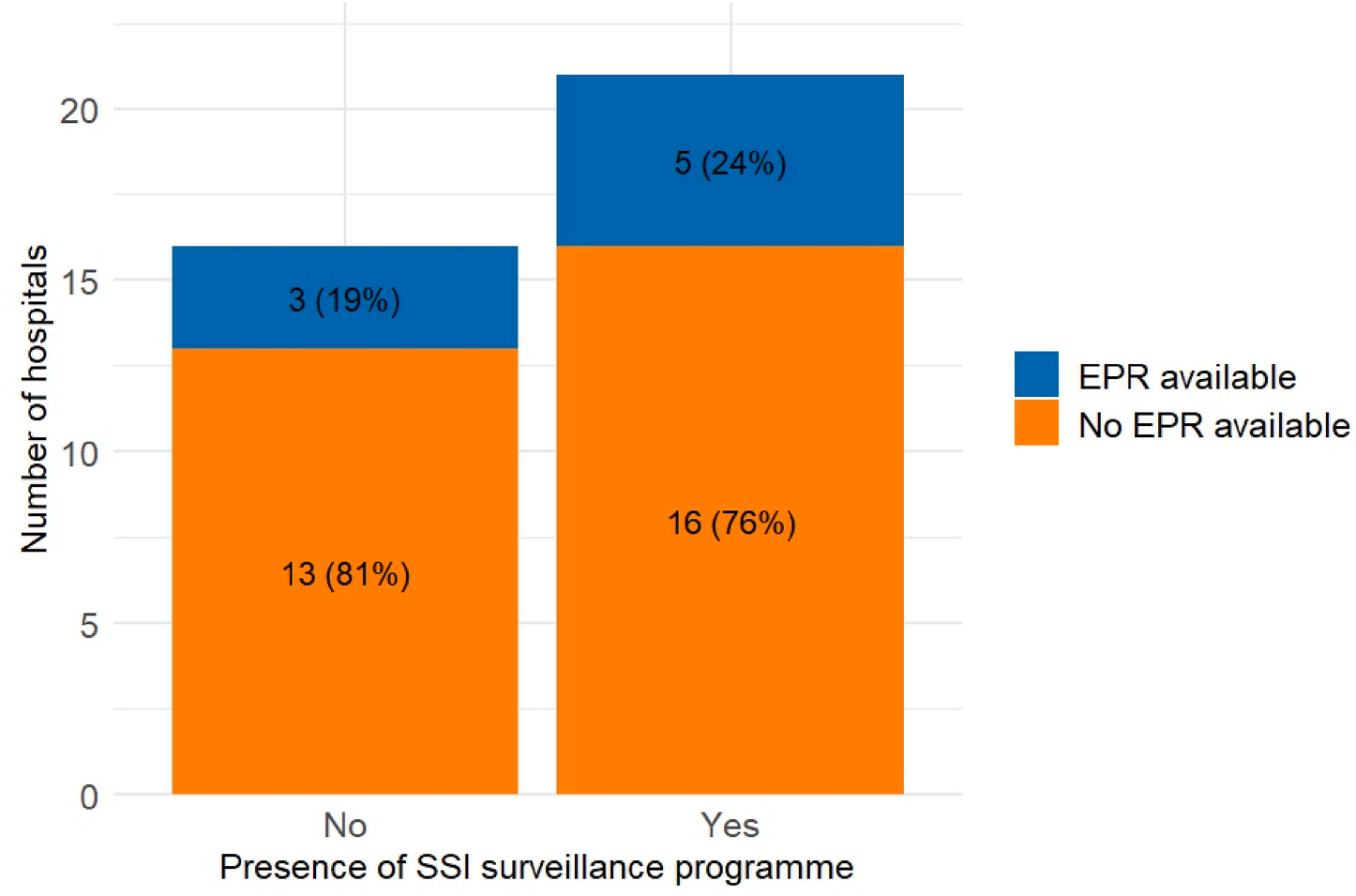
EPR availability by SSI surveillance programme status. EPR, electronic patient record; SSI, surgical site infection.

Respondents identified significant challenges in implementing and maintaining SSI surveillance. Programmes were often resource-intensive and manual, relying on individual staff for data collection. Staffing shortages and limited IT infrastructure contributed to variability in surveillance approaches. Dedicated SSI personnel were strongly associated with programme presence (17/21 [81%] vs 0/16 [0%]; Fisher’s exact *P*<0.001; Figure 2), underscoring the need for protected staffing and funding. Electronic patient record availability was limited and did not differ between hospitals with and without programmes (5/21 [24%] vs 3/16 [19%]; Fisher’s exact *P*=1.0; Figure 3), with few systems integrating theatre, laboratory, and clinical data. As a result, surveillance frequently required manual review of records and data transfer. Upgrading IT systems to enable automated data extraction and integration would improve accuracy and sustainability, as echoed in international reports [3].

Postdischarge surveillance remains a key challenge, and this survey highlights the current resource-intensive nature of this work. A previous Irish study reported that 90% of caesarean section SSIs were diagnosed post hospital discharge [8]. This variation has direct implications for case ascertainment: 11 of 21 programmes (52%) relied on passive, readmission-based detection (9/21) or undertook no postdischarge surveillance (2/21), thereby capturing only infections severe enough to prompt readmission and likely underestimating true incidence relative to active follow-up. With increasingly shorter hospital stays and the potential for infection to manifest up to 90 days postoperatively, effective postdischarge surveillance is essential. Without it, the true burden of SSIs is likely to be significantly underestimated. Digital tools enabling remote wound monitoring and patient self-reporting show promise for enhancing postdischarge surveillance [9,10].

Limitations of this survey include its voluntary nature, potential response bias, and the absence of independent verification of reported practices. Invitations were directed to clinical microbiology consultants, who are typically involved in SSI surveillance through hospital governance structures; however, this approach may not capture the perspectives of all stakeholders and inclusion of a broader multi-disciplinary cohort would have strengthened the findings.

As Ireland progresses towards a national SSI surveillance programme, this survey demonstrates substantial local activity despite resource constraints. A co-ordinated national approach should prioritise standardisation of definitions and methodologies while acknowledging existing diagnostic uncertainty and variation in local capacity. International models offer useful guidance. In England, for example, the UK Health Security Agency (UKHSA) SSI Surveillance Service mandates surveillance of defined orthopaedic categories using standardised case definitions and postdischarge follow-up across participating trusts [3]; such comprehensive systems enable detailed benchmarking but are resource-intensive, whereas targeted approaches, including case-only reporting, may be more feasible in constrained settings. A hybrid model, allowing flexible participation aligned with local capability, may be most appropriate, similar to the ECDC unit-based and patient-based framework [2].

Within this context, a tiered approach provides a pragmatic framework for implementation. At a minimum level, hospitals could undertake targeted surveillance of high-priority procedures using standardised definitions. An intermediate model could incorporate prospective surveillance with structured postdischarge follow-up. At an optimal level, comprehensive surveillance across multiple procedures could be supported by integrated electronic systems, enabling benchmarking. Integration with established national audit structures, similar to the IHFD/NOCA model, could support phased implementation, governance, and data quality.

Regardless of the approach, investment in staffing, digital infrastructure, and governance will be essential to support integration and sustainability. Leveraging existing local expertise will facilitate development of a cohesive, high-quality national surveillance system.

## CRediT authorship contribution statement

**E. Glynn:** Writing – original draft, Visualization, Project administration, Methodology, Investigation, Formal analysis, Data curation, Conceptualization. **S. Hurley:** Writing – review & editing, Visualization, Validation, Formal analysis. **L. Webster:** Writing – review & editing, Software, Formal analysis, Data curation. **C. Rock:** Writing – review & editing, Supervision. **K. O'Connell:** Writing – review & editing, Supervision, Conceptualization. **S. O'Donnell:** Writing – review & editing, Supervision, Conceptualization. **E. Brannigan:** Writing – review & editing, Supervision, Conceptualization.

## Ethics statement

Formal ethical approval was not required in accordance with institutional policy. The survey collected no patient-level personal data, and responses were analysed and reported in aggregate to maintain institutional anonymity. Participation was entirely voluntary and informed consent was implied through completion of the survey.

## Funding sources

None declared.

## Conflict of interest statement

None declared.
